# Addressing disruptions in childhood routine immunisation services during the COVID-19 pandemic: perspectives from Nepal, Senegal and Liberia

**DOI:** 10.1136/bmjgh-2021-005031

**Published:** 2021-07-06

**Authors:** Sameer M Dixit, Moussa Sarr, Daouda M Gueye, Kyle Muther, T Ruston Yarnko, Robert A Bednarczyk, Adolphus T Clarke, Fatoumata Diakhite, Aliou Diallo, Bonheur Dounebaine, Shankar B Duwadi, Anna S Ellis, Nancy Fullman, Nathaniel Gerthe, Jhalak S Gautam, Kyra A Hester, Gloria Ikilezi, Rokhaya S Mbengue, Souleymane Mboup, Birahim P Ndiaye, Rajesh Man Rajbhandari, David E Phillips, Matthew C Freeman

**Affiliations:** 1 Center for Molecular Dynamics Nepal, Kathmandu, Nepal; 2 Institut de Recherche en Santé de Surveillance Epidémiologique et de Formations, Dakar, Senegal; 3 Last Mile Health, Monrovia, Liberia; 4 Rollins School of Public Health, Emory University, Atlanta, Georgia, USA; 5 Expanded Programme on Immunisation, Republic of Liberia Ministry of Health, Monrovia, Liberia; 6 Focal Point for EPI and Surveillance Activities, Health Center of Rufisque, Dakar, Senegal; 7 Expanded Programme on Immunisation Unit, WHO Country Office Senegal, Dakar, Senegal; 8 Health Section Chief, Benighat, Nepal; 9 Gates Ventures, Kirkland, Washington, USA; 10 Child Health and Immunization Section, Family Welfare Division, Department of Health Services, Ministry of Health and Population, Kathmandu, Nepal; 11 Focal Point for EPI and Surveillance Activities, Health Center, Philip Maguilene Senghor of Yoff, Dakar, Senegal

**Keywords:** immunisation, vaccines, health services research, child health, COVID-19

**Summary box**

- While routine immunisation (RI) was among the health services most affected during the earlier phases of the COVID-19 pandemic, country programmes employed various mitigation strategies to maintain vaccine delivery and/or resume interrupted programming.
- Perspectives from Nepal, Senegal and Liberia highlight six key components of addressing COVID-19’s effects on RI during the earlier phases of the pandemic: (1) prioritising continued services with strengthened infection prevention control; (2) engaging in effective communications and mobilisation activities, especially to offset misinformation about COVID-19 and vaccines; (3) identifying alternative locations and approaches to providing vaccine services (eg, conducting door-to-door vaccination if facility-based services were not possible); (4) instituting infection prevention controls and physical distancing measures (and adapting service provision accordingly); (5) setting up systems and strategies for reaching children who missed doses amid periods of disruption; and (6) conducting catch-up campaigns as soon as SARS-CoV-2 transmission risks can be minimised.
- The ways in which COVID-19 has affected RI services have varied over time and across settings, underscoring the importance of contextually tailored mitigation efforts and adaptation, given evolving challenges amid an ongoing pandemic.
- As countries roll out COVID-19 vaccines, it will be vital to avoid one-size-fits-all implementation strategies and to support the continuance of RI services through this next phase of COVID-19 response.

## Introduction: Synthesising Lessons Learned For Addressing COVID-19 Effects on Routine Immunization (RI) through the Exemplars in Global Health Partnership (EGH)

RI emerged as one the most disrupted health services in early-2020 to mid-2020,^1^ a global systems shock associated with the COVID-19 pandemic. In May 2020, an estimated 80 million children were thought to be affected by interrupted vaccination services and campaigns,^2^ and over 60% of 105 countries reported at least partial RI disruptions to WHO.^1^ If left unaddressed, these disruptions are poised to halt or even reverse decades of global progress achieved in vaccine delivery and child health.^3 4^

Yet with great challenges can come great opportunity: knowledge and strategies gained from navigating pandemic’s effects on service delivery could help pave the ways for innovation, adaptions and resilience in the ways that RI programmes are approached more broadly.

How countries have sought to address pandemic-related disruptions has undoubtedly—and rightfully—varied. The complexities of vaccine delivery and how they intersect with sociocultural mores, political commitment and technical requirements make locally relevant, tailored approaches fundamental to any successful RI programme. This is particularly true as COVID-19 continues to affect countries in different ways over time, a trend further fuelled in 2021 by unequitable COVID-19 vaccine roll-out and surges of new virus variants. Lessons learnt during earlier pandemic phases may help support health service continuity and adaptations throughout 2021, a challenge we will collectively face until SARS-CoV-2 is fully contained worldwide.

With this commentary, we discuss experiences from Nepal, Senegal and Liberia in addressing COVID-related disruptions of RI services in early-2020 to mid-2020. We represent a range of stakeholders brought together through the EGH partnership, an initiative focused on identifying lessons learnt from positive outliers in global health and harnessing the expertise of programme leaders, researchers and funders to better understand what underpins health gains and implementation success.^5–7^ As such, our perspectives may not be shared by others in RI programmes for a given context; further, they may not represent challenges and/or mitigation strategies occurring now.

## Main types of pandemic-related effects on RI and approaches to mitigation

The COVID-19 pandemic affected key determinants of vaccination—facility readiness, intent to vaccinate and community access^8 9^—as well as how these drivers interact together to support timely, effective vaccine services worldwide (table 1). On their own, each of these disruptions could negatively impact vaccination rates; in combination, they formed substantial obstacles to RI programmes as a whole.

**Table 1:** Summary of main types of pandemic-related disruptions for childhood RI, by vaccination driver, with global examples and reports from Nepal, Senegal and Liberia

Vaccination driver		
Facility readiness	Intent to vaccinate	Community access
Vaccination driver definition		
Health system supply and capacity, via health facilities, to adequately meet the demand of patients who seek vaccine services	Demand for vaccine services by caretakers that, in the absence of all other barriers, would result in children being vaccinated	Ability to carry out vaccination via barriers and facilitators between facility readiness and vaccination intent
Examples of documented disruptions related to COVID-19		
Delayed shipments of vaccine stocks and key supplies due to travel restrictions and reduced flight availability.^18^ Reduced or halted service availability and/or hours of operation. Inadequate PPE, sanitation and infection control supplies. Deployment of staff for COVID-19 response.^19^ Staff shortages due to COVID-19 infections, fear of exposure and/or burnout.	Fear of virus exposure at healthcare settings.^6^ Individuals instructed to postpone or delay non-urgent healthcare services due to concerns about overburdening healthcare facilities. Misinformation or rumours on COVID-19 and vaccination safety.	Postponement or cancellation of mass vaccination campaigns, as well as new vaccine introductions.^2^ Restrictions on movement outside of residences and physical access to vaccine services due to social distancing policies and lockdown measures enacted to curb viral transmission.^2^
Country-reported types of disruptions (via Exemplars in Global Health^5^)		
Nepal		
50% of the country’s immunisation service centres ceased operations after lockdown measures were enacted at the end of March 2020 (ie, from 16 000 to 8000). Hospitals, inclusive of their outpatient departments, shut down frequently from COVID-19 cases among healthcare professionals.		Nepal halted measles-rubella campaign in mid-April 2020 due to safety concerns.
Senegal		
Health centre staff were tasked with COVID-19 management and response, reducing their availability for other services.	Patient attendance substantially declined for vaccine services, and some caretakers refused care when home visits were offered by community workers in full PPE. Rumours spread about an ‘eventual evil’ COVID-19 vaccine trial in the country.	School closures halted school-based human papillomavirus service delivery. Mass gatherings were prohibited in mid-March 2020, postponing community-based outreach activities and mobilisation.
Liberia		
Health facility-based outreach activities were suspended. Initial gaps in PPE availability for staff occurred.	Growing vaccine hesitancy took place, fuelled by rumours of COVID-19 vaccines being tested on citizens.	Early days of the country’s state of emergency and curfew were associated with declines in the uptake of RI services.

Vaccination driver definitions are adapted from the Phillips and colleagues’ framework on determinants of vaccination.^8 9^ Reported pandemic-related disruptions in Nepal, Senegal and Liberia are based on information provided by coauthors via direct correspondence and responses provided through guided interviews (online supplemental file 1).

EPI, Expanded Programme on Immunization; PPE, personal protective equipment; RI, routine immunisation.

10.1136/bmjgh-2021-005031.supp1 Supplementary data

In Nepal, about 50% of immunisation centres ceased operations during the initial onset of lockdown measures at the end of March 2020; furthermore, cases among healthcare workers would cause clinics to abruptly suspend services. In mid-April 2020, a nationwide measles–rubella campaign was suspended due to safety concerns. Measles outbreaks occurred in 4 of Nepal’s 77 districts, which were thought to be related to such disruptions.

For Senegal, with mass gatherings prohibited in mid-March 2020, vaccination activities and mobilisation efforts led by Badiènou Gokh and other community-based health workers had to be postponed. Human papillomavirus (HPV) vaccine delivery was halted as schools, Senegal’s main platform for HPV vaccination, closed. At facilities, health worker availability became constrained by concurrent deployment for COVID-19 management and response. Senegal also saw patient attendance fall, which was attributed to a combination of movement restrictions, concerns about COVID-19 exposure, and/or rumours about COVID-19 and vaccine reliability.

In Liberia, growing vaccine hesitancy emerged as a major challenge, with rumours rapidly spreading about COVID-19 vaccines being tested on citizens. Concerned about potential longer-term consequences of vaccine misinformation, Liberia temporarily halted facility-based outreach services. Uptake of RI services also declined during the earlier days of Liberia’s state of emergency, while health facility staff also faced inadequate supplies of personal protective equipment (PPE).

These represent only a fraction of RI disruptions experienced in 2020,^1 2 4 10^ underscoring the importance of swiftly implementing catch-up activities and closing gaps in coverage. WHO has issued recommendations on providing immunisation services during the pandemic,^11–14^ with its guidance evolving alongside world’s evidence base on COVID-19 and how to weigh the relative risks of SARS-CoV-2 transmission against the costs of service disruption. In late March 2020, for countries with surging infections and without active measles or polio outbreaks, WHO recommendations viewed exposing healthcare workers and community members to the novel coronavirus as greater than temporarily postponing mass campaigns.^11^ As the world’s understanding of COVID-19 improved and the magnitude of service disruptions began to surface,^2^ WHO issued follow-up guidance on implementing mass vaccination campaigns^12^ and principles for maintaining or resuming RI.^13 14^

How countries have then acted—adapting such guidelines for local needs or developing their own mitigation strategies against COVID-19 disruptions based on prior experience or some combination of each—has become an area of great interest. Drawing from experiences in Nepal, Senegal and Liberia, as well as desk research, we identified six main components to approaches for addressing pandemic-related effects on RI (table 2).

**Table 2:** Main mitigation components for addressing COVID-19 effects on RI services, reports from Nepal, Senegal, and Liberia and additional country examples

COVID-19 mitigation component	Country-reported approaches (via Exemplars in Global Health^5^)			Additional examples of country approaches to addressing COVID-related disruptions to RI
	Nepal	Senegal	Liberia	
Prioritise continued immunisation services at both national and local levels.	Nepal government and Ministry of Health and Population supported uninterrupted RI service, even during lockdown periods. Nepal introduced the rotavirus vaccine in July 2020, which occurred during a national lockdown period.	Facility-based RI services were sustained, even as community-based outreach and mobilisation services were temporarily suspended during gathering bans.		In Laos, RI services were conducted at fixed sites with physical distancing during national lockdown.^2^ In Afghanistan, efforts to maintain RI services included strong engagement with religious and community leaders and trainings on vaccine-preventable disease surveillance for volunteers.^20^
Conduct effective communications and outreach efforts to combat misinformation and engender trust in COVID-19 guidance and RI efforts.		Media campaigns and broadcasting were conducted through cable channels, local TV channels and radio spots to emphasise the importance of childhood immunisation, particularly as EPI programmes were scaling up mobilisation efforts.	Liberia’s EPI developed and implemented multistage risk communication strategic action plan to address widespread rumours and vaccine hesitancy identified through a vaccine perception study in Montessrado and Margibi counties.	Virtual campaigns were conducted with Paraguay’s President and Minister of Health via social media.^21^ In Cambodia, mobile teams of community health workers with long-standing ties to communities were dispatched for targeted outreach services.^15^ Sri Lanka’s Ministry of Health leveraged mass media to inform populations that essential health services had resumed, including vaccination, and urged community members to bring children to clinics.^22^
Identify alternative locations, infrastructure and service delivery platforms outside of traditional health clinics.	In districts with measles outbreaks, 94 ORI centres were set up to facilitate faster catch-up and response, aiming to ensure ORI centres were within a 30 minute walk for the majority of populations.	Health workers offered alternative hours for vaccine administration (eg, after work, during the weekends) in collaboration with community-based organisations. Community health workers conducted house-to-house vaccination for HPV services amid continued closures of schools.		In Brazil, vaccination posts were set up in empty locations due to lockdown measures (eg, schools).^21^ Drive-through and home-based vaccination services were conducted in Brazil, Chile and El Salvador.^21^ In Bolivia, ‘immunisation brigades’ were deployed to high-risk populations at nursing homes and jails.^21^
Institute IPC and physical distancing measures, and adapt service provision practices accordingly.	Nepal government developed PPE guidelines and ensured all vaccinators received masks and eyewear, and full PPE whenever possible (ie, gloves, footwear, and overalls in addition to masks and eyewear). Formal IPC training occurred and distancing measures were implemented prior to field mobilisation when measles–rubella campaigns resumed in June 2020. Nepal’s EPI implemented maximum safety and infection control during rotavirus introduction in July 2020.	Ministry of Health and Social Actions adapted WHO guidelines and gave instructions to all regions and health centres for implementation, allowing staff to tailor these guidelines to align with local needs and strategies across health system levels. Facility staff implemented IPC protocols, established physical distancing measures and procured PPE, and ran or participated in training webinars coordinated with national-level programmes to prepare for continued immunisation service delivery.	Drawing from WHO guidance, Liberia’s EPI developed country-specific IPC guides and implemented training at facilities for vaccinators to support safe administration practices and reduce exposure risk. Working with country partners, the EPI team ensured expanded access at least basic PPE, strengthening both the protection of vaccinators and trust among individuals seeking services at facilities and/or being met by vaccinators through community outreach campaigns.	In Pakistan, polio vaccine workers were trained to administer vaccines without touching children, and were provided with full PPE. 23 The Solomon Islands introduced rotavirus vaccine via a phased province-by-province approach rather than the centralised introduction model, advising people not to travel outside of their home provinces to reduce transmission risk.^24^ In Uganda, government cars were respurposed as shuttles to drive patients and caregivers to clinics to access health services including immunisations.^20^ In Sri Lanka, patients were limited to one child or family per hour for immunisation services, and sought to give multiple catch-up vaccinations and/or vaccinate all children in a given family per visit when possible.^15 22^ In Paraguay, tents were set up outside of health centres to separate people seeking vaccination from patients who are ill.^21^ In Ethiopia, a mass measles campaign targeting 14.4 million children was extended for a longer time period to limit crowding, and conducted vaccinations in outside, well-ventilated areas with physical distancing and sanitation protocols.^25^
Set up systems to track missed doses—and target follow-up efforts—in areas with the largest disruptions.		Based on contact records kept at district offices, facility staff texted or called parents whose children missed immunisation visits.		In Karachi, Pakistan, children who missed doses were identified and their parents were sent text messages about service resumption via the city’s Electronic Immunisation Registry mobile application.^26^ In Ghana, parents of children who missed vaccination visits were called about catch-up services and scheduling home visits as needed.^27^
Conduct catch-up campaigns as soon as SARS-CoV-2 transmission risks can be effectively mitigated.	In Nepal, a mass measles–rubella vaccination campaign was resumed in June 2020 after activities were halted in mid-April due to safety concerns.^15^		In Liberia, ‘periodic intensification of RI’ (ie, campaign-style vaccine services) were conducted in at least 11 districts by August 2020. RI services began to resume alongside multistage risk communications plan *and* expanded safety protocols.	Postponed polio vaccination campaigns were resumed with extensive PPE and safety protocols in several countries, including Syria in June 2020,^15 28^ Burkina Faso^29^and Pakistan^23^ in July 2020, among others. In Zambia, the June 2020 Child Health Week was leveraged to include catch-up campaigns for inactivated polio vaccines and HPV vaccination.^24^

Reported approaches to mitigating to pandemic-related disruptions in Nepal, Senegal and Liberia are based on information provided by coauthors via direct correspondence and responses provided through guided interviews (online supplemental file 1). Additional examples were identified through desk research conducted from June to September 2020.

EPI, Expanded Programme on Immunization; HPV, human papillomavirus; IPC, infection prevention control; ORI, outbreak response immunisation; PPE, human papillomavirus; RI, routine immunisation.

First, prioritising the continuity of RI services, at both national and local levels, was a key component. This was particularly evident in Nepal, where the Ministry of Health and Population directed its Family Welfare Division to implement safety protocols and to adapt service provision as necessary to maintain RI services amid the country’s extended lockdown period (ie, end of March through most of July 2020).

Second, implementing communications and outreach activities were vital for addressing misinformation and strengthening trust in both COVID-19 guidance and RI activities. Such work was the cornerstone to Liberia’s mitigation approach, with a vaccine perception study identifying myths about COVID-19, limited trust in both new and old vaccines, and delays in vaccination timeliness as the primary barriers to uptake. In collaboration with key partners, Liberia’s Expanded Programme on Immunization (EPI) then developed a multistage communications plan, mapping key messages to target audiences, communication channels, and impact metrics for short-term, medium-term and long-term objectives.

Third, identifying alternative delivery options beyond health clinic settings was critical for promoting service continuity during the COVID-19 pandemic. For instance, Senegal’s RI programme began offering vaccine sessions at alternative hours, including after work and during weekends, and community health workers conducted home visits for HPV vaccination after school-based services became unavailable amid widespread closures.

Fourth, instituting IPC and distancing measures while modifying service provision practices has been fundamental for maximising the safe continuance or resumption of vaccination services. Nepal, Senegal and Liberia each adapted WHO guidelines to national and local settings, conducted IPC trainings and procured PPE for vaccinators.

Fifth, leveraging current—or setting up—systems to track missed doses and target catch-up efforts remains crucial for RI recovery efforts. In Senegal, healthcare workers have been using contact records maintained at district offices to text or phone parents whose children missed immunisation visits. In the absence of such information, short-term delays in completing vaccination schedules could quickly turn into more persistent coverage gaps and higher risk for vaccine-preventable disease.

Sixth, conducting catch-up RI services and campaigns as soon as SARS-CoV-2 transmission risks could be mitigated is imperative to addressing pandemic-related disruptions. For instance, Nepal resumed its nationwide measles–rubella mass campaign in June 2020 after a 1.5-month pause,^15^ and by August 2020, Liberia had conducted periodic intensification of RI in at least 11 districts.

Not every approach was used or perhaps needed in each country, just as COVID-19 has affected RI services in different ways across settings. For instance, Liberia’s EPI had to weigh the potential consequences of rapidly deteriorating vaccine confidence and trust against temporarily suspending facility-based outreach services in April 2020. Their pivot to identifying obstacles to vaccine uptake and establishing their risk communications strategy before resuming services again involved distinct planning and implementation approaches from those required elsewhere. In Nepal, large numbers of people would still show up at immunisation centres to vaccinate their children during lockdown periods; while high vaccine acceptance and service-seeking typically would be welcome, during the ongoing COVID-19 pandemic, such turn-out could heighten potential virus exposure and transmission risk in the absence of clear distancing measures and IPC protocols. As such, Nepal had to carefully coordinate immunisation sessions to maximise safety, especially when the rotavirus vaccine was introduced in July 2020. For Senegal, adapting modes of RI delivery was central to mitigating COVID-related effects on immunisation, especially when typical RI platforms—community-based outreach and school-based services—were not viable during nationwide gathering restrictions and closures. In response, community health workers have gone house-to-house for vaccination visits and have sought to provide greater flexibility in visit timing. What unites these diverse settings and programmes is the recognition that each level of vaccination barriers or facilitators must be addressed, from patient acceptance and trust to community access; the willingness to adapt to and test alternative approaches; and the overarching dedication to providing RI to all.

It is worth noting that pandemic-related impacts on RI may not be wholly known for months or even years to come. Similarly, the longer-term success of past and present efforts to maintain or resume RI remains unclear; this is particularly true as epidemic trajectories continue to change and now many countries are rapidly deploying COVID-19 vaccines alongside new virus variants and corresponding surges. Going forward, it is crucial to better understand implementation dimensions of modified RI services, including acceptability, feasibility, costs and effectiveness,^16 17^ especially as EPI programmes consider if and how to resume prepandemic operations. As more data are collected, analysed and disseminated, through the EGH partnership, we hope to continue collating lessons learnt about programmatic strategies and innovations that countries implemented to sustain vaccine delivery during the COVID-19 crisis.

## Conclusions

The COVID-19 pandemic has threatened progress made in child health worldwide, posing widespread disruptions and challenges to RI programmes, healthcare providers and patients alike. Lessons learnt through this global crisis are only as beneficial as efforts to collaborate and rethink approaches to promoting resilience, equity, and sustainability for both RI programmes and preparedness more broadly. The world now faces its next reckoning with COVID-19: deployment of COVID-19 vaccines amid ongoing, or even surging, SARS-CoV-2 transmission. How we collectively approach RI services and vaccine campaigns, from local to global levels, will ultimately determine how the remainder of this pandemic will unfold.

## Data Availability

All data relevant to the study are included the article.
