# Supplementary material for: Addressing disruptions in childhood routine immunisation services during the COVID-19 pandemic: perspectives from Nepal, Senegal and Liberia
Source: BMJ Glob Health. 2021 Jul 6;6(7):e005031. doi: 10.1136/bmjgh-2021-005031 (PMC8264163; doi:10.1136/bmjgh-2021-005031)
Supplement: Supplementary data [file bmjgh-2021-005031supp001.pdf]

Effect of COVID-19 on the Delivery of Routine Immunizations

AREA OF EXPLORATION

How has COVID-19 impacted the delivery of routine immunizations? What steps has your country taken to mitigate these challenges?

BACKGROUND

The COVID-19 pandemic has substantially affected routine immunization services in countries around the world. To minimize the negative effects of these disruptions, countries are working to maintain or resume key immunization services. Our hope is to learn the strategies and steps that individual countries are taking, so that these best practices can be shared more broadly.

The appendix of this document contains specific areas we are hoping to learn more from you about. Thank you in advance, and please let us know if you have any questions.

APPENDIX

Areas we are hoping to learn more about – *Please track who is interviewed on condition that they have consented and document any relevant quotes.*

i. At the national level, have specific guidelines been developed, and if so, what are they and how have they been implemented?

ii. To what degree has COVID-19 disrupted the routine immunization program at both national and subnational levels (~March through August)? What factors have been affected?

Examples could include:

Example	Detail
Vaccine stocks/Delivery processes	*Reported challenges and issues in vaccine procurement and shipments*
Reduced mobility following imposed lockdowns or fear of getting exposed to COVID	*Could result in decreases in demand for vaccines and decreased uptake of vaccines, along with decreases in clinic attendance*
Facility closures	*Reduced or closed operations of health clinics/mobile operations; could be influenced by insufficient PPE or infection control equipment/supplies, lockdown measures and restrictions on within-country movement, parental/caregiver concerns about virus*
Staffing norms	*Health worker shortages due to redeployment for COVID-19 response and/or concerns about virus exposure*

Resource allocation	*Disruptions in financing obligations/mechanisms*
Outreach activities	
Planned campaigns or SIAs	*Postponing previously planned vaccine introductions or mass campaigns*

iii. How is the country tracking each of these?

iv. What existing aspects of the immunization system have helped it meet the challenges posed by COVID? (Note: please record any detail on drivers of resilience in the health system, where resilience refers to the level of preparation and ability to withstand shocks to the health system)

v. What other programs or policies have been implemented to address the ongoing disruptions? Examples could include:
- Communication strategies
- HRH optimization, task shifting
- Repurposing resources (financial or otherwise) towards response activities
- New stakeholder engagements
- Alternative vaccination or outreach locations
- Clinic, outreach, or campaign scheduling changes
- Other operational strategies

vi. Looking ahead, for both the short-term and long-term, how do you think these actions to address COVID will affect immunizations and the health system as a whole?

vii. Have the existing guidelines from the global community (e.g. from WHO, UNICEF, Gavi) provided enough detail for decision-making? What additional input, guidance, or other materials would be helpful?
